# The role of YKL-40 in the pathogenesis of autoimmune diseases: a comprehensive review

**DOI:** 10.7150/ijbs.67587

**Published:** 2022-05-21

**Authors:** Kalthoum Tizaoui, Jae Won Yang, Keum Hwa Lee, Ji Hong Kim, Minseok Kim, Sojung Yoon, Yeonwoo Jung, Joon Beom Park, Kitae An, Hyeok Choi, Donggyu Song, HyunTaek Jung, Seongmin Ahn, Taeho Yuh, Hee Min Choi, Jae Ha Ahn, Younjuong Kim, Sanghyun Jee, Hyeongsun Lee, Soohwa Jin, Jun-Gu Kang, Bohyun Koo, Joo Yeop Lee, Kyoung Min Min, Wonseok Yoo, Hyeong Jun Rhyu, Yeonjung Yoon, Min Ho Lee, Sung Eun Kim, Jimin Hwang, Ai Koyanagi, Louis Jacob, Seoyeon Park, Jae Il Shin, Lee Smith

**Affiliations:** 1 Laboratory Microorganismes and Active Biomolecules, Sciences Faculty of Tunis, University Tunis El Manar, Tunis, Tunisia.; 2 Department of Nephrology, Yonsei University Wonju College of Medicine, Wonju, Republic of Korea.; 3 Department of Pediatrics, Yonsei University College of Medicine, Seoul, Republic of Korea.; 4 Yonsei University College of Medicine, Seoul, Republic of Korea.; 5 Department of Epidemiology, Johns Hopkins Bloomberg School of Public Health, Baltimore, USA.; 6 Parc Sanitari Sant Joan de Deu/CIBERSAM, ISCIII, Universitat de Barcelona, Fundacio Sant Joan de Deu, Sant Boi de Llobregat, Barcelona, Spain.; 7 ICREA, Pg. Lluis Companys 23, Barcelona, Spain.; 8 Faculty of Medicine, University of Versailles Saint-Quentin-en-Yvelines, Montigny-le-Bretonneux, France.; 9 The Cambridge Centre for Sport and Exercise Sciences, Anglia Ruskin University, Cambridge, UK.

**Keywords:** YKL-40, Autoimmune disease, Pathogenesis, Diagnostic marker, Biomarker

## Abstract

YKL-40, a chitinase-3-like protein 1 (CHI3L1) or human cartilage glycoprotein 39 (HC gp-39), is expressed and secreted by various cell-types including macrophages, chondrocytes, fibroblast-like synovial cells and vascular smooth muscle cells. Its biological function is not well elucidated, but it is speculated to have some connection with inflammatory reactions and autoimmune diseases. Although having important biological roles in autoimmunity, there were only attempts to elucidate relationships of YKL-40 with a single or couple of diseases in the literature. Therefore, in order to analyze the relationship between YKL-40 and the overall diseases, we reviewed 51 articles that discussed the association of YKL-40 with rheumatoid arthritis, psoriasis, systemic lupus erythematosus, Behçet disease and inflammatory bowel disease. Several studies showed that YKL-40 could be assumed as a marker for disease diagnosis, prognosis, disease activity and severity. It is also shown to be involved in response to disease treatment. However, other studies showed controversial results particularly in the case of Behçet disease activity. Therefore, further studies are needed to elucidate the exact role of YKL-40 in autoimmunity and to investigate its potential in therapeutics.

## 1. Introduction

YKL-40 is a 40-kDa heparin-and-chitin binding glycoprotein and also known as chitinase-3-like protein 1 (CHI3L1) 1, a 38-kDa heparin-binding glycoprotein 2 or human cartilage glycoprotein 39 (HC gp-39) 3. YKL-40, coded by the gene CHI3L1 in chromosome 1q31-q32. 4,5, was first discovered in 1992 in the secretion of human osteoblastic cells in culture 6. The name YKL-40 was derived from the three N-terminal amino acids - tyrosine (Y), lysine (K), and leucine (L) - present on the secreted form 7. Although YKL-40 is a member of glycoside 18 family of chitinases 8, it does not have enzymatic properties 5,9,10.

It is expressed and secreted by various kinds of cells such as chondrocytes, fibroblast-like synovial cells, vascular smooth muscle cells, and macrophages. Its exact pathophysiological function and mechanism of action is currently unknown, but it is thought to play a role in inflammation, tissue remodeling, and angiogenesis 11. It is involved in the activation of the innate immune system, extracellular matrix remodelling, 8,12 and the differentiation of CD14+ monocytes to CD14- and CD16+ macrophages 13. YKL-40 stimulates the proliferation of human connective tissue cells (fibroblasts, chondrocytes, synovial cells) in a dose-dependent manner 14,15 and also plays important roles in the antigen-induced T-helper 2-response, antigen sensitization and IgE induction as well as activation of innate immune cells 16. Recent studies have suggested important roles of YKL-40 in various autoimmune diseases. However, these findings have not been reviewed comprehensively. This review will discuss the potential of YKL-40 in the pathogenesis of autoimmune and rheumatic diseases, such as rheumatoid arthritis, psoriasis, systemic lupus erythematosus, Behçet disease, and inflammatory bowel disease. Possible therapeutic strategies by targeting YKL-40 will also be discussed.

## 2. Rheumatoid arthritis (RA) and YKL-40

Rheumatoid arthritis (RA) is a chronic autoimmune disease principally affecting the synovial joints, causing pain and limitation of motion by the destruction of arthritic cartilage and joint ankylosis. The main aim of RA treatment is to achieve early remission with minimal disease activity. Disease modifying anti-rheumatic drugs (DMARDs) (i.e. sulfasalazine, methotrexate and hydroxychloroquine), glucocorticoids (GC) and biologics such as anti-tumour necrosis factor (TNF)-α inhibitors have been used in the treatment of RA. Disease activity is usually assessed by some indices, such as the 28-joint disease activity score (DAS28) 17 which evaluates the count of tender and swollen joints, inflammation and patient´s assessment of the disease activity.

Although the exact mechanism of joint destruction is still elusive, the presentation of exogenous and autologous antigens by antigen presenting cells (APCs) to T-cells are considered to be an early process of disease, and YKL-40 has been recognized as a potential candidate autoantigen 2,3,4,5,7. Recently, there have been publications on important roles of YKL-40 in the pathogenesis of RA such as correlations with disease activity of RA and diagnostic or prognostic biomarkers. We will discuss the role of YKL-40 in RA pathogenesis as well as its potential therapeutic target.

A total of 47 articles discussing the association between RA and YKL-40 were identified from PubMed search. There were two review articles: one about the association between YKL-40 and RA 18, and the other about the association between YKL-40, inflammation, and cancer 19. Twenty-three articles were reviewed in these two review articles 14,15,16,20-40. From 21 articles newly published after the review, one article was excluded due to multiple confounding variables 41, and another was excluded because it was a phase 1 clinical trial which did not discuss the effect of YKL-40 42. We have summarized the key findings of 43 eligible articles (**Table 1**), 24 of which were included in reviews by Johansen et al. 18 and Kzhyshkowska et al. 19 which have been published thereafter.

### 2.1 Correlation between YKL-40 and pathogenesis of rheumatoid arthritis

YKL-40 is an important autoantigen in RA immunopathology. Several researches showed locally elevated concentration of YKL-40 in serum and synovial fluid of RA patients, suggesting that YKL-40 plays an important role in RA pathophysiology 43-45. The immune reaction induced by YKL-40 differs, showing anti-inflammatory phenotypes in healthy people versus proinflammatory phenotypes in half of the RA patients 20. mRNA for YKL-40 was identified specifically in human articular chondrocytes and liver 21 and overexpressed in the synovium and peripheral blood mononuclear cells (PBMCs) from RA patients 14. YKL-40 expression is closely related to differentiation of monocytes into macrophages which produce higher levels of YKL-40 in rheumatoid synovium 15. YKL-40 binds to the HLA-DR4 peptide-binding motif which leads to mononuclear cell proliferation 16,22. Intracellular HLA-DM also has a crucial role in presenting YKL-40 to CD4+ T cell through “epitope editing” 23. Moreover, the APCs presenting the immunodominant epitope of YKL-40 mainly appeared at the primary disease sites 24. One phase 1 study reported that HLA-DR4B1-Org3660l, a peptide derived from YKL-40, inactivated T cell response and induced immune tolerance in RA patients 25. It also indicates that YKL-40 has an association with RA pathogenesis.

**Figure 1:**
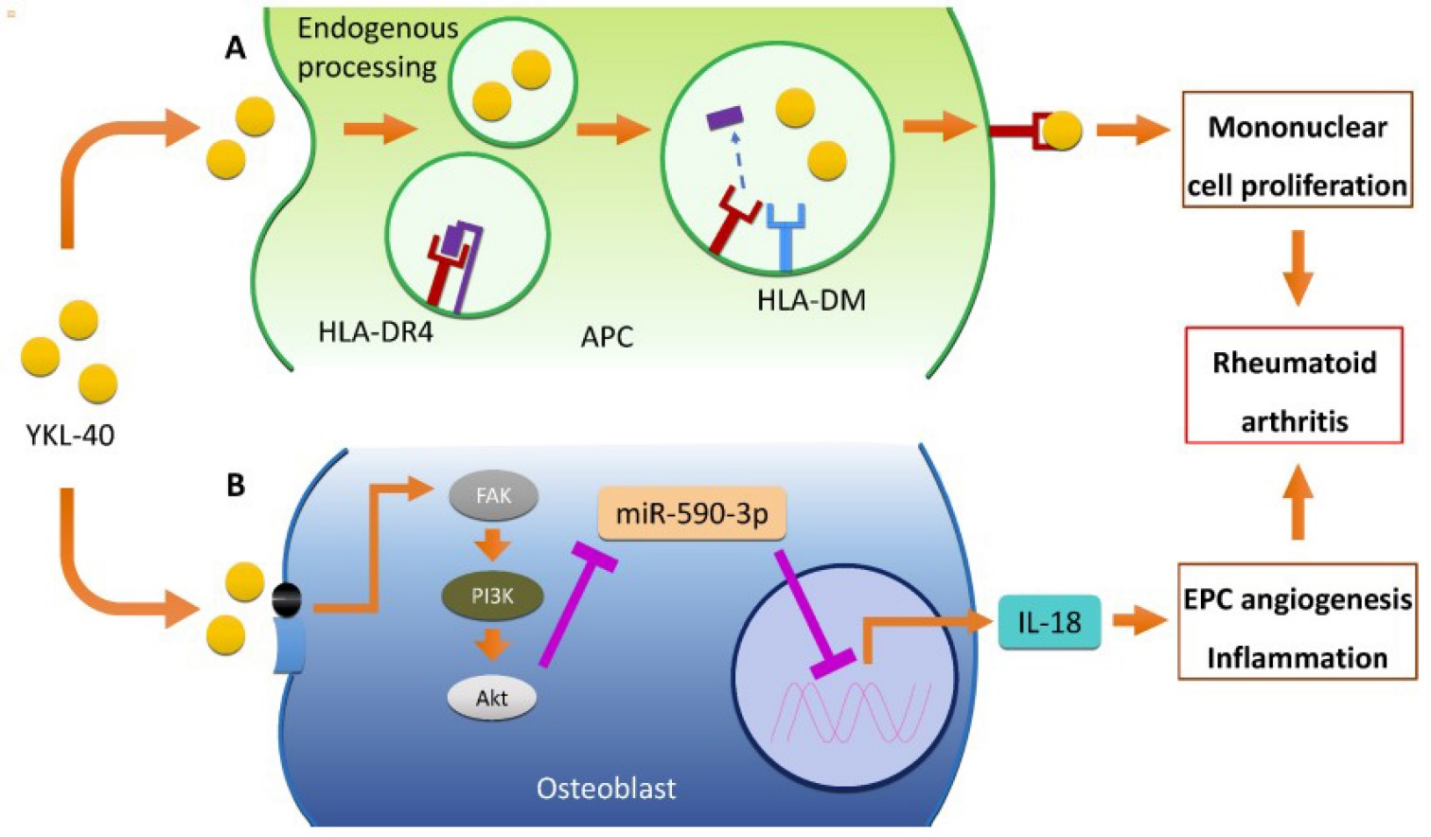
Role of YKL-40 in RA pathogenesis. (A) YKL-40 binds to the HLA-DR4, inducing mononuclear cell proliferation through HLA-DM dependent MHC II-mediated presentation of APCs. Intracellular HLA-DM also plays an important role in the presentation of YKL-40 to CD4+ T cells via "epitope editing”. YKL-40 induces mononuclear cell proliferation by binding to the HLA-DR4 peptide binding motif (B) YKL-40 also activates FAK/PI3K/Akt pathway in osteoblast, resulting in IL-18 production and EPC angiogenesis through inhibition of miR-590-3p. This in turn stimulates endothelial progenitor cell (EPC) angiogenesis, promoting inflammation, a process critical in the pathogenesis of RA. Abbreviations: RA: rheumatoid arthritis; HLA: human leukocyte antigen; MHC: major histocompatibility complex; APC: antigen presenting cell; FAK: focal adhesion kinase; PI3K: phosphoinositide 3-kinase; Akt: protein kinase B; IL-18: interleukin-18; EPC: endothelial progenitor cell; miR: microRNA.

Endogenous processing and major histocompatibility complex (MHC) II-mediated presentation of YKL-40, play major roles in the pathophysiology of RA 46. Human ex-vivo differentiated DR4+ dendritic cells and macrophages, which were similar to synovial joint APC, were also capable of MHC II-presentation of YKL-40 epitopes 44. A study using binding assay visualized the constitution of HLA-DR4 (B1*0410)/YKL-40 complex and showed that this can modify antigen-specific, pro-inflammatory responses in HLA-DR4 transgenic mice 45. Not only inducing T cell proliferation, YKL-40 also activates the FAK/PI3K/Akt pathway, thereby inducing the production of IL-18 in osteoblasts and the inhibition of miR-590-3p. This, in turn, stimulates endothelial progenitor cell (EPC) angiogenesis, subsequently promoting inflammation, a process critical to the pathogenesis of RA 47. Serum and synovial YKL-40 levels were strongly correlated with the levels of other inflammatory cytokines like IL-1β and TNF-α, suggesting that these molecules together play an important role in RA pathogenesis and disease activity 48. Moreover, Petersson et al. (2006) also reported that arginine-vasopressin (AVP) and parathyroid hormone-related peptide (PTHrP) stimulated YKL-40 secretion particularly in RA chondrocytes 26,32, while Nielsen et al. suggested the association between the g.-131 (C > G) promoter SNP in the *CHI3L1* gene and the serum concentration of YKL-40 49. Proposed pathogenic mechanisms of YKL-40 in RA are demonstrated in **Figure 1**.

### 2.2 Efficacy of YKL-40 as a biomarker of rheumatoid arthritis

#### Diagnostic marker

Since the serum and synovial fluid YKL-40 levels in RA patients were higher than those in the healthy controls, YKL-40 seemed to be a useful diagnostic marker for RA 16,27. In one study which synovial biopsy samples are obtained from 154 patients, presentation of specific YKL-40 peptides in the context of class Ⅱ MHC was a highly specific histopathologic marker with a PPV of >90% for atypical RA patients 50.

However, mere serum or synovial YKL-40 level has a significant limitation to be an effective diagnostic tool since it cannot differentiate RA from other joint inflammatory diseases 28. Production of YKL-40 might be a just response to an altered tissue environment, since YKL-40 plays a role in cartilage-remodeling process 16. Moreover, some argued that YKL-40 values have a limited efficacy as a diagnostic marker. Harvey et al. (2000) suggested that serum YKL-40 level does not provide any additional information that would not be attainable by means of conventional biochemical measurements of disease activity like ESR or CRP levels 29. Sekine et al. (2001) also suggested that it is not a sensitive marker for RA diagnosis as YKL-40 was detected in only 1 of 87 RA patients 30.

In contrast to mere expression of YKL-40, monoclonal antibody 12A staining in the synovial membrane was highly specific for RA. Monoclonal antibody 12A, which acts directly against HLA-DR4/HC gp‐39^263-275^ complex, can be a novel immune-pathologic diagnostic tool for RA 24. Steenbakkers et al. showed that monoclonal antibody 12A has the highest specificity for the complex among 5 monoclonal antibodies that bind to HLA-DR4/HC gp‐39^263-275^ complex, and therefore can be used to effectively detect MHC/peptide/TCR complexes in the synovium of RA patients 31. Also, in review paper by Johansen et al., the author suggested YKL-40 as a potential diagnostic marker of RA because of higher YKL-40 elevation in RA rather than OA compared to healthy people 18. However, there are few papers about YKL-40 as a diagnosis marker. Therefore, whether YKL-40 can be used to diagnosis in RA should be further studied.

#### Correlation with disease activity

There may be various criteria for determining disease activity, such as DAS28, inflammation, radiological score (Larsen score), functional disability, erosion which means joint destruction, and clinical course. In order to explain the relationship between YKL-40 and disease activity, it is necessary to explain whether or not there is a significant relationship with YKL-40 level in each criterion. In addition, whether or not YKL-40 is a marker of disease activity also depends on whether it is a baseline value or a value that reflects treatment response. In fact, Table 1 summarizes many studies explaining the relationship between YKL-40 by distinguishing the baseline state from the treatment response state.

The association between YKL-40 and RA disease activity have been studied primarily via the measurement of YKL-40 levels in the serum (sYKL-40). sYKL-40 levels are increased significantly in RA patients compared to healthy controls 16,32-33, inactive RA groups 16, or in inactive patients who developed active RA 34. YKL-40 levels have also been studied in the synovium, which have shown an increase of YKL-40 levels in the synovial fluid 35 or an increase in the number of YKL-40 positive cells in the synovial membrane 27. Correlations have been found between disease activity and sYKL-40 levels in RA patients 27,36, and even between disease activity and the immune response to YKL-40 levels in vitro 37.

In one study, sYKL-40 level was positively correlated with radiological scores used to assess joint destruction, but not with joint pain or swelling 32. Similarly, YKL-40 expression in the synovial tissue was reported to have correlation with joint destruction 14.

Only one paper was an in vitro experiment 51. The other papers were in vivo experiments 43,50,52-57. One of the seven papers evaluated the correlation between sYKL-40, cf-PWV and IMT-C as well as DAS28 54. Also, one of the seven papers evaluated sYKL-40 and synovial thickening and vascularization scores 55. In the early phase of GPI-induced arthritic mice study, high sYKL-40 was detected 51. In two randomized controlled trial (RCT) studies, sYKL-40 level had a positive correlation with early rheumatoid arthritis (ERA) disease activity 52,53. In a cross-sectional study including 42 ERA patients and 35 healthy patients, sYKL-40 levels were highly correlated with DAS28 54. Another study including 51 ERA patients and 21 polyarthritis (PA) patients, increased YKL-40 in serum was observed only in RA patients 56. In one study where 25 Danish RA patients got repeated measurement of various serum markers during treatment, YKL-40 serum was significantly elevated in RA patients 43. In one study, the MBDA score where twelve markers including serum YKL-40 are combined was significantly correlated with RA disease activity 50. Interestingly, a study evaluating arterial stiffness by cf-PWV and IMT-C by carotid ultrasonography 54 showed that sYKL-40 was highly correlated with cf-PWC and IMT-C as well as DAS28. These results suggest the possibility of early detection of atherosclerosis using sYKL-40 54. Correlation between YKL-40 level and synovial thickening and vascularization score was observed in one study 55. Therefore, YKL-40 serum could be used as a disease activity marker of RA.

Seven papers are available for evaluating sYKL-40 level during RA treatment 34,35,38,43,53,56,58. sYKL-40 levels decreased with RA treatment in several studies 34,35,38,43,53,58. However, there were also conflicting results 58. Administration of intra-articular glucocorticoids, DMARD therapy, and prolonged TNF-alpha neutralization led to a significant decline in sYKL-40 levels in RA patients 34,35,38. DMARD therapy in combination with prednisolone was reported to be more effective than DMARD therapy alone when the decrease in sYKL-40 levels was compared between the two treatment groups after 1, 7, 14, and 30 days 34. Specifically, in one RCT study where 99 ERA patients received DMARD therapy for 26 weeks, sYKL-40 levels decreased with DMARD 53,58. In a 1-year pilot study where 20 RA patients received infliximab and concomitant methotrexate therapy for 52 weeks, sYKL-40 levels decreased after infliximab therapy 58. In one study including 25 Danish RA patients, sYKL-40 significantly decreased after anti-TNF-α agents 43. However, in another study, sYKL-40 level was decreased only in patients who achieved remission, but not in all of RA patients 56. Therefore, sYKL-40 could be used to evaluate treatment response.

##### Correlation with long-term prognosis

One study showed that sYKL-40 levels have a correlation with RA progression (assessed by the Larsen score and the extent of bone erosions) 32, while other studies suggested no association between sYKL-40 and RA progression 36,39. Four papers using YKL-40 as a marker are available for long-term prognosis of RA. The others evaluated YKL-40 as a long-term prognosis marker in RA patients 52,58,59. Four papers had a consensus that sYKL-40 seemed to be not a long-term prognosis marker and to be not associated with radiographic progression in RA 18,52,58,59. In one cohort study where 238 RA patients are followed for 10 years, sYKL-40 level didn't show any correlation in 5- and 10-years radiographic progression 59. In 1-year pilot study where 20 RA patients got infliximab and concomitant methotrexate therapy for 52 weeks, YKL-40 did not show correlation with radiographic joint destruction 58. Also, in two investigatory-initiated RCTs on naïve ERA patients, YKL-40 was not useful in predicting patient's prognosis in radiographic or clinical aspects 52. Therefore, YKL-40 did not seem to be a long-term prognosis marker, nor was it associated with radiographic progression in RA.

### 1.3 Effect of YKL-40 suppression in rheumatoid arthritis

Three papers evaluating YKL-40 suppression are available for RA treatment 51,60,61. Injecting YKL-40 in an early phase of GPI-induced arthritis mice caused decreased antigen-specific T cell proliferation and cytokine production 51. By using surgical procedures, a study investigated the relationship between and intra-nasal YKL40 injection in lymph nodes and tolerance induction in mice 61. They concluded that lymph nodes that drain the nasal mucosa is crucial in tolerance induction in mouse model AG (OVA) 61,67. This showed the possibility of injecting YKL-40 as RA treatment 51,61. However, in a phase II, double-blinded RCT study where RA patients received internal YKL-40 therapy, there was no significant change in DAS28 compared to placebo group 60. Therefore, other strategies including changes in way to convey YKL-40 or combination of other immune regulators should be studied.

## 3. Psoriasis and YKL-40

Psoriasis is an immune-mediated systemic inflammatory disease, characterized by erythematous, well-demarcated plaques with silvery scale 62. Psoriasis could be classified into five different types: plaque, guttate, inverse, pustular, and erythrodermic psoriasis. Plaque psoriasis, also referred as psoriasis vulgaris, is the most prevalent type. Pustular psoriasis and erythrodermic psoriasis are rare but can be life-threatening. There are diverse comorbidities related to psoriasis, including psoriatic arthritis, cardiovascular disease, lymphoma and Crohn's disease 63. Psoriasis has high prevalence and demonstrates chronic progress without a definite cure, making it more challenging to deal with. Through PubMed search, a total of 11 studies were found to discuss the relationship between psoriasis and YKL-40 (chitinase-3-like protein 1, CHI3L1, human cartilage glycoprotein-39) **(Table 2)** 64-74. Most of them showed a positive association between psoriasis and YKL-40 64-72, while 2 studies reported negative or no association 73,74.

### 3.1 Positive correlation between psoriasis and YKL-40

Multiple biomarkers have been used for the diagnosis of psoriatic diseases. Increase in white blood cell count, C-reactive protein (CRP), and several cytokines such as IFN-γ were found in patients with psoriasis; however, these markers alone lack sensitivity and specificity for evaluating the severity of disease 75. Several studies recently showed the possibility of YKL-40 as a new diagnostic marker for psoriasis and its possible correlation with disease severity. Imai Y et al. first observed the elevation of YKL-40 in patients with psoriasis vulgaris compared with control group, and the difference was more obvious in patients with generalized pustular psoriasis, a more severe inflammatory form of psoriasis 64. sYKL-40 level was also suggested to show positive relationship with the severity of skin lesions in patients with psoriatic arthritis, a type of arthritis that occurs in psoriasis patients 65. Ahmed S et al. stated that sYKL-40 level might be used for evaluating disease activity and angiogenesis in patients with psoriasis 66. YKL-40 could also be helpful for diagnosing and monitoring patients with psoriatic arthritis as an inflammatory marker 67, 68. Salomon et al. stated that an increased YKL-40 level reflects the presence of systemic inflammation rather than cutaneous lesions 67 and found its correlation with the severity of psoriatic arthritis, which was scored with 28-joint Disease Activity Score (DAS-28) in the study 68. There were conflicting findings regarding YKL-40's legitimacy in evaluating treatment response. Baran A et al. stated that YKL-40 might be interpreted as a marker of psoriasis, but not for evaluating metabolic condition or efficacy of treatment 69. However, decreased sYKL-40 level after narrow-band ultraviolet B phototherapy was observed in psoriasis vulgaris patients 70.

Furthermore, sYKL-40 level is also related to vascular defects in psoriasis patients. YKL-40 level is positively correlated with carotid intima-media thickness and defective aortic elasticity in patients with psoriasis 71. According to Erfan G et al, YKL-40 might be associated with endothelial dysfunction in psoriasis and could be applied for managing cardiovascular diseases in high-risk psoriasis patients 72.

### 3.2 Negative or no correlation between psoriasis and YKL-40

There are two studies that show negative or none correlation between psoriasis and YKL-40. Ataseven et al. reported that the YKL-40 levels of psoriasis group are not significantly different from the levels of healthy controls 73. Jensen et al. revealed that there is no correlation between YKL-40 and psoriasis severity 74. However in psoriatic arthritis group, which is a type of arthritis that occurs in psoriasis patients, YKL-40 level had association with disease severity and treatment response 74.

## 4. Systemic lupus erythematosus (SLE) and YKL-40

SLE is an autoimmune disease that involves multiple organs including the skin, kidney, brain, and joints 76. Majority of patients are women of childbearing age, which accounts for 90% of total SLE patients 77. There are several classic markers that have been used to estimate disease activity in SLE patients, such as anti-dsDNA titer and measurements of C3, C4, CH50 78. A total of 3 associations between SLE and YKL-40 were identified from a PubMed search (**Table 3**) 36,79,80.

Two studies which compared YKL-40 levels between SLE patients and healthy controls showed concordant findings. Vos et al. first revealed that SLE patients have higher YKL-40 levels than healthy controls 36. However, YKL-40 levels in SLE patients were lower compared to RA patients and did not correlate with disease activity 36. Wcisło et al. also showed that average plasma levels of YKL-40 is about twice higher in the SLE group than in controls 79. Likewise, YKL-40 had no correlation with disease activity or the severity of joint involvement 80. There was an in vitro study that showed reduced reactivity of T cells from SLE patients in response to YKL-40 80. Vos et al. incubated T-cells in the settings of five types of YKL-40 and compared its growth rate between different disease entities including SLE 80. They studied the responses of T-cells to YKL-40 in patients with various inflammatory conditions 80 and found that T-cells from RA patients showed a proliferative response to YKL-40 80. In contrast, T-cells from SLE patients showed low response to YKL-40 80.

## 5. Behcet's disease (BD) and YKL-40

BD is a systemic inflammatory disorder with recurrent oral ulcers, genital ulcers, eye lesions, and skin lesions 81. It can also involve joints, central nervous system, gastrointestinal tract, and large vessels 81. Although the pathogenesis of BD is not fully known, neutrophilic hyperactivity and overproduction of pro-inflammatory cytokines and reactive oxygen species (ROS) are considered to be major mechanism of the disease 81,82,83. PubMed search identified two studies describing the association between BD and YKL-40 (**Table 4**) 84,85.

Seo et al. first reported the correlation between BD and YKL-40 84. By comparing sYKL-40 levels of control group and inactive/active BD patients, they revealed that YKL-40 was significantly higher in BD patients 84. In addition, patients in the active BD group showed an elevation in sYKL-40 levels compared with inactive BD patients 84. Since YKL-40 levels showed a positive correlation with disease activity, they proposed YKL-40 as an alternative marker to monitor disease activity in BD patients 84. Bilen et al. also reported YKL-40 elevation in BD patients 85. However, this study failed to show the association between YKL-40 levels and disease activity, which is inconsistent with the previous study 85. Therefore, further research is required to clarify the correlation between YKL-40 and BD activity.

## 6. Inflammatory bowel disease (IBD) and YKL-40

A total of 14 associations between inflammatory bowel disease and YKL-40 were identified from a PubMed search (**Table 5**) 83-96.

### 6.1 Correlation between YKL-40 and disease activity in inflammatory bowel disease

A link between YKL-40 and the activity of IBD lies on the process of fibrosis and inflammation during the natural course of IBD 86. Ten papers reported increased levels of YKL-40 associated with the severity of the disease 86-95. In both Crohn's disease and ulcerative colitis, the sYKL-40 levels were significantly correlated with C-reactive protein level and disease activity 86. In Crohn's disease, sYKL-40 level was shown higher in patients with intestinal strictures than in those without intestinal strictures 87. Vind et al. found that sYKL-40 level is increased in 40-50% of ulcerative colitis and Crohn's disease patients with active disease, and the level was also increased in 30% of patients with Crohn's disease which is clinically inactive 88. Ytting H et al. reported a case where s YKL-40 level is increased as an appearance of Sweet's syndrome in a patient previously diagnosed with ulcerative colitis, sYKL-40 level acted as a marker of disease activity of Sweet's syndrome 89. Increased sYKL-40 level was also discovered as the marker for demonstrating articular involvement in inflammatory bowel disease 90. Punzi L et al. discovered a similar result showing sYKL-40 level was only increased in IBD patient with arthritis, IBD patients without arthritis showed no difference from healthy controls 94. Among the peptides derived from YKL-40, the human cartilage glycoprotein-39 ^263-275^ level was increased in IBD 91. The fecal YKL-40 level was found to be a marker to assess endoscopic ulceration level in IBD 89. The fecal sYKL-40 level was also correlated with the severity and disease activity in IBD 93. In Crohn's disease, sYKL-40 is an autoantigenic target, IgA and secretory IgA antibodies against sYKL-40 level can serve as a marker facilitating the serological diagnosis 95.

### 6.2 Correlation between YKL-40 and development of cancer in inflammatory bowel disease

The YKL-40 expression in colonic epithelial cells is a biomarker for neoplastic changes in IBD 95. In a mouse model, the YKL-40 induced cell proliferation and survival while involving down-regulation of the pro-apoptotic S100A9 protein, which promoted tumorigenic changes in the colon and led tumor cells survive and proliferate 97. In another mouse model, colonic epithelial cells with high levels of YKL-40 expression exhibited malignant transformation *in vivo* when exposed to azoxymethane, a well-known colonic carcinogen 98. Higher expression of the YKL-40 was found in the distal colon compared to the proximal colon in mice, resulting in higher chances of tumorigenesis in the distal colon 99.

## 7. YKL-40 as biomarker of other human diseases with autoimmune mechanism

The serum YKL-40 levels were higher in the multiple sclerosis (MS) group than in control, and which levels in the patients with relapsing remitting multiple sclerosis (RRMS) were correlated with the patients' expanded disability status scale (EDSS) scores and ages. No relationships were determined between the serum YKL-40 levels and the other variables. The findings from this study suggested that YKL-40 may be a useful marker for the inflammatory process of MS 100. Serum levels of YKL-40 were significantly higher in 40 female patients with systemic sclerosis compared to 14 healthy female controls. In contrast, miR-214 expression in plasma from SSc patients was significantly downregulated compared to controls which shows that the binding of YKL-40 and miR-214 is involved in the mechanism of inflammation and fibrosis 101. YKL-40 was higher in the poorly controlled symptom and exacerbation group and in patients with non-atopic asthma compared with stable asthma. YKL-40 appears to increase in proportion to the degree of inflammation in diseases with an immune mechanism 102. Compared to neurological controls, increased YKL-40 levels were detected in sCJD and Alzheimer's disease (AD) but not in vascular dementia (VaD) or in dementia with Lewy bodies (DLB)/Parkinson's disease dementia (PDD). Further, two independent patient cohorts were used to validate the increased CSF YKL-40 levels in Creutzfeldt-Jakob disease (sCJD). YKL-40 is a disease-specific marker of neuroinflammation showing its highest levels in prion diseases 103.

## 8. Concluding remarks and future perspectives

The biological function of YKL-40 glycoprotein, also known as chitinase-3-like protein 1 (CHI3L1) 1 or human cartilage glycoprotein 39 (HC gp-39) 3, is not that clear, but it is speculated to have some connection with inflammatory reactions and autoimmune diseases 9,11. We reviewed 51 articles that discussed the association of YKL-40 with RA, Psoriasis, SLE, BD and IBD. Results highlight the value of YKL-40 as biomarker of autoimmunity and rheumatic diseases.

In the first place, we found 21 articles discussing associations between RA and YKL-40. Two pathogenesis of RA related to YKL-40 are unknown; acting as an autoantigen or an inducing factor of IL-18 expression 44-47. There were two articles including a review article on using YKL-40 as a diagnostic marker of RA 18, 50. Both showed the possibility of YKL-40 as diagnostic marker of RA, and one of them demonstrated the presentation of specific YKL-40 peptides in the context of MHC-II 50. As a result, YKL-40 could be assumed as a diagnostic marker of RA, but further studies are needed. There were nine articles investigating the correlation of RA disease activity with YKL-40 18,43,52-57, all of which demonstrated a correlation between YKL-40 level and disease activity of RA. Furthermore, one of the papers that used cf-PWV and IMT-C as disease activity indices presented the potentiality of YKL-40 as an early detection marker of atherosclerosis in RA patients 54. Including a review article, there were five articles on evaluating s YKL-40 level during RA treatment 18,43,53,56,58. They revealed YKL-40 levels decreased after therapy by DMARD, infliximab or anti-TNF-alpha agents. Notably, in one of those studies, YKL-40 level was decreased only in patients that achieved remission, but not in all RA patients 56. As follows, YKL-40 could be used to evaluate treatment response. We could find four articles examining the availability of YKL-40 as long-term prognosis of RA, including a review article 18,52,68,59. But there was no evidence at all that sYKL-40 level is associated with radiographic progression and long-term prognosis. Lastly, three papers are available for RA treatment using YKL-40 suppression 51,60,61. This protocol had some controversy, so further studies are warranted.

There were 11 studies on relation between psoriasis and YKL-40. Five of them imply YKL-40 could be used for evaluating disease activity because YKL-40 level is elevated in a more severe form of inflammation than in a less severe form or in controls and in systemic inflammation rather than in cutaneous lesions 64-68, though Jensen et al. argued that there is no correlation 73. Furthermore, there were two articles which demonstrate YKL-40 level is also related to vascular defects in psoriasis patients 72. Two of them investigated YKL-40's legitimacy in evaluating treatment response, but their results were inconsistent. There were two studies showing no correlation of sYKL-40 level with presence of disease and psoriasis severity 74. However, in the psoriatic arthritis group, YKL-40 level had an association with disease severity and treatment response 74.

Two studies which compared YKL-40 levels between SLE patients and healthy controls showed concordant findings 36,79. They revealed that SLE patients had higher YKL-40 levels than healthy controls 79, but YKL-40 level had no correlation with disease activity or the severity of joint involvement 79. There was an in vitro study that showed reduced reactivity of T cells from SLE patients in response to YKL-40 80.

Two studies describing the association between BD and YKL-40 were identified 84,85. Two studies revealed that YKL-40 was significantly higher in BD patients 82. But it was controversial whether YKL-40 level is related to BD activity 84,85.

Ten papers reported increased level of YKL-40 associated with the severity of IBD 86-95. Also, in the papers, YKL-40 level is increased in active disease, patients with Sweet's syndrome, or those with articular involvement 86-90,94. Not only sYKL-40 level, but also HC gp-39 ^263-275^ level, fecal YKL-40 level, IgA and secretory IgA level against sYKL-40 seemed to have potential to be used as markers 91-93,95. In addition, the YKL-40 expression in colonic epithelial cells could be a biomarker for neoplastic changes in IBD 96. YKL-40 down-regulate the pro-apoptotic S100A9 protein, which promoted tumorigenic changes in colon and led tumor cells survive and proliferate 97. Higher expression of YKL-40 in colonic epithelial cells led to higher chances of tumorigenesis 98,99.

## 9. Conclusion

In conclusion, this systemic review indicates that YKL-40 is a very promising molecule as a biomarker of RA both in diagnostic and therapeutic aspects. Further studies on YKL-40 for autoimmune diseases as Psoriasis, SLE, BD, IBD and other diseases with immune mechanism are essential to fully elucidate its clinical significance and utility.

### Consent for publication

All the authors checked and gave their approval of this version to be published.

### Funding

No financial support was provided for research conduct and/or preparation of the article.

### Author contributions

All authors made substantial contributions to all of the following; (1) conception and design of the study, data acquisition, or analysis and interpretation of data; (2) drafting or critical revision of the article for intellectual content; and (3) final approval of version to be submitted.

## Figures and Tables

**Table 1:** List of studies on the relationship between RA and YKL-40 (chitinase-3-like protein-1, human cartilage glycoprotein-39).

Authors, year	Study design	Main study findings
van Bilsen JH et al, 2004 20	-PBMCs obtained from RA patients were stimulated in vitro with HC gp-39. -Elispot analyses were used to analyze the production of cytokines.	• HC gp-39 may have anti-inflammatory phenotype in healthy individuals. • gp-39 immune response in RA patients has pro-inflammatory phenotype.
Hakala BE et al, 1993. 21	-HC gp-39 was isolated using PCR and western blotting. -Northern blotting and RT-PCR was used to detect HC gp-39 mRNA both in synovial membranes and in cartilage acquired from patients with RA.	• Purified and sequenced HC gp-39 has regions similar to three other mammalian secretory proteins. • HC gp-39 mRNA was present in articular chondrocytes.
Baeten D et al, 2000 14	-Immunohistochemistry and flow cytometry were used to study expression of HC gp-39 in synovium and PBMCs. -Synthesis and secretion of HC gp-39 were evaluated by RT-PCR and ELISA.	• HC gp-39 expression was more significant in RA patients compared to spondyloarthropathy patients and healthy controls. • gp-39 level is correlated with the degree of joint destruction in RA.
Kirkpatrick RB et al, 1997 15	-In situ hybridization was used to detect HC gp-39 mRNA in macrophages obtained from ST of 5 RA patients.	• HC gp-39 is expressed in primary human macrophages. • Its expression is closely related to differentiation of monocytes into macrophages in RA patients.
Harvey S et al, 1998 16	-Sandwich-type ELISA was used to compare serum Chondrex (HC gp-39) in healthy controls and in arthritis groups.	• Chondrex (HC gp-39) values are highly increased in active RA patients compared to healthy controls and inactive RA groups. • Chondrex levels may be an effective marker in estimating RA disease activities. • By assessing Chondrex values of RA patients, the effectiveness of the DMARD therapy can be identified.
Verheijden GF et al, 1997 22	-Self-reactive peptides within HC gp-39 were tested for their ability to induce mononuclear cell responses in RA patients or healthy donors' peripheral blood. -Native HC gp-39 was injected into mouse model to study its ability to develop arthritis.	• HC gp-39-derived motif-based peptides were selectively recognized by peripheral blood T cells from RA patients. • HC gp-39-derived motif-based peptides are associated with the development of a chronic, relapsing arthritis.
Patil NS et al, 2001 23	-Antigen presentation assays using DR*0401-restricted T cell hybridomas made by transgenic mice with HC gp-39.	• HLA-DM has a crucial role in presenting HC gp-39 to CD4+ T cell hybridomas. • HLA-DM dependent pathway has inference for HC gp-39 presentation in RA.
Baeten D et al, 2004 24	-Immunostaining with monoclonal antibody 12A on synovial joints captured from RA patients and healthy controls.	• HC gp-39 is correlated with histologic characteristics of inflammation in RA joints. • Monoclonal antibody 12A staining can be a novel immune-pathologic diagnostic tool for RA.
Kavanaugh, A et al, 2003 25	-31 persistent RA patients with positive HLA-DRB1*0401 were randomized to 7 infusions of AG4263 for 6 weeks.	• AG4263 is a soluble complex of Org36601 which is derived from HC gp-39. • Infusion of AG4263 with methotrexate was safe and well tolerated for persistent RA disease activity without generalized immunosuppression.
Petersson et al., 2006 26	-Analysis of cultured chondrocyte samples from RA patients, OA patients, and healthy subjects.	• Both AVP and PTHrP increase YKL-40 secretion in RA chondrocytes. • AVP decreases YKL-40 secretion in healthy and OA chondrocytes. • AVP and PTHrP influence RA pathogenesis as proinflammatory hormones.
Volck et al., 1998 27	-Immunoelectron microscopy and immunohistochemistry studies on neutrophils and bone marrow cells.	• Synovial fluid of active RA patients contains high levels of YKL-40 as well as abundant neutrophils, which are thought to be important in joint destruction. • YKL-40 released from neutrophils may play a role in tissue remodeling or degradation in various inflammatory diseases including RA.
Vos et al., 2000 28	-Comparison of plasma HC gp-39 levels in 50 RA, 51 OA, 24 SLE, 26 IBD patients and 49 healthy controls by one-way ANOVA.	• RA, OA, SLE, and IBD patients have significantly higher serum levels of HC gp-39 than healthy controls.
Harvey et al., 2000 29	-Analysis of sYKL-40 by ELISA in 57 ERA patients over 19 months	• sYKL-40 does not provide any additional information that would not be attainable by means of conventional biochemical measurements of disease activity.
Sekine et al., 2001 30	-ELISA assay and western blotting of serum samples YKL-39 and YKL-40 from 87 RA and 47 SLE patients.	• YKL-39 and YKL-40 share more than 50% amino acid and nucleotide sequence homology. • YKL-39, unlike YKL-40, which is speculated to be an autoantigen in RA, does not seem to be a sensitive marker for RA diagnosis. • The immune response to YKL-39 was shown to be independent of that to YKL-40.
Steenbakkers PG et al, 2003 31	-Monoclonal antibodies that bind DRαβ1*0401/HC gp-39(263-275) complexes were used to investigate the MHC-Ag complexes, and expression of HC gp-39 was studied in ST of DRαβ1*0401-positive RA patients by immunohistochemistry.	• The HC gp-39(263-273) epitope is specifically essential for binding of MHC protein. • Among 5 mAb studied, mAb 12A has the most specificity for DRαβ1*0401/HC gp-39(263-275) complexes, and therefore mAb 12A was used to detect MHC/peptide/TCR complexes in the synovium of RA patients.
Matsumoto et al., 2001 32	-Assessment of sYKL-40, sIGF-I, and sIL-6 levels in 72 RA patients and 40 healthy subjects measured by ELISA.	• RA patients had significantly higher sYKL-40 levels than the healthy controls. • sYKL-40 levels were positively correlated with sIL-6 and CRP levels but negatively correlated with sIGF-I. • sYKL-40 levels correlated with radiological score and severity of functional disability but not with joint pain.
Johansen et al, 2001 33	-Analysis of sYKL-40 samples from RA patients treated with DMARDs for 36 months measured by ELISA.	• sYKL-40 levels in ERA patients were significantly correlated with radiological progression determined by the Larsen score • sYKL-40 may provide information independent of CRP on disease activity.
Johansen et al., 1999 34	-1-year longitudinal study of sYKL-40 obtained from 156 RA patients by RIA.	• In RA, sYKL-40 levels correspond to disease activity: levels decreased significantly in active patients who became clinically inactive, while levels increased in inactive patients who developed active RA.
Volck et al., 2001 35	-Analysis of serum and synovial fluid YKL-40and other biochemical markers determined by RIA and ELISA.	• YKL-40 was detected in the synovial membrane of RA and OA patients. • The number of YKL-40 positive cells in the inflamed synovial membrane is positively correlated with the severity of inflammation. • Intra-articular glucocorticoid injection was followed by a decline in sYKL-40 levels.
Vos K et al, 2000 36	-Growth with 5 different HC gp-39 derived peptides followed by measuring multiplication of PBMC as well as recording disease activity score in RA, SLE, IBD, OA and healthy controls.	• In autoimmune diseases including RA, HC gp-39 derived peptides are the objects of T cell immunity. • Especially HC gp‐39 derived peptide 259-271 are known to be correlated with disease severity in RA.
den Broeder A et al, 2002 37	-Univariate and multivariate analyzes were used to assess the association between serum markers of radiological progression and cartilage and synovial membrane turnover (HC gp-39). -Integrated measures of disease activity.	• Long term TNF-alpha neutralization decreased the levels of HC gp-39, in RA patients.
Peltomaa et al., 2001 38	-Analysis of sYKL-40 in 52 early onset RA patients by ELISA during a 2-year prospective follow-up	• Baseline sYKL-40 levels prior to anti-rheumatic therapy were significantly higher in ERA patients compared to healthy controls. • sYKL-40 is an inflammatory marker that correlates with disease activity, but does not have predictive value concerning radiographic progression or clinical course.
Combe B et al, 2001 39	-A cohort study of 191 RA patients conducted for three years.	• The average odds ratio of YKL-40was 1.3(p=0.5) in predicting radiographic progression.
Fusetti F et al, 2003 40	-Analysis of crystallized structures of HC gp-39 by using the hanging drop vapor-diffusion method with recombinant HC gp-39.	• HC gp-39 is apparently eligible autoantigen of RA and though its exact physiological function is unexplained. • HC gp-39, epitopes 259-271 and 263-275 are associated with disease activity. • Even if region 266-275 leads to the protein surface, residues 259-265 placed in the near-by of the chitin-binding groove.
Knudsen et al., 2009 43	-Repeated measurements of pIL-6, pVEGF, and sYKL-40 for 25 Danish RA patients during treatment.	• In treatment of RA, 18 of 25 patients had significantly decreased pIL-6, pVEGF, and sYKL-40 which were significantly elevated before treatment.
Tsark EC etval., 2002 44	-Blood monocyte-derived dendritic cell and macrophage were incubated with native human CII, HC gp39, and synovial fluid from RA patients. and the comparison with T cell hybrids produced by immunizing DR4-transgenic mice with CII 259-263 and HC gp39^263-275^ peptides.	• Human ex vivo differentiated dendritic cell and macrophage that were incubated with synovial fluid from RA patients differentially presented CII and HC gp39 MHC II epitopes. • CII and HC gp39 might be important autoantigens in RA immunopathology and show different mechanisms of their presentation by dendritic cell and macrophage.
Boots AM et al., 2007 45	-Competition binding assay was used to map epitopes that strongly bind to T cell hybridomas obtained from HC gp-39-immunized HLA-DR4 transgenic mice.	• Anchor variant peptide can modify antigen-specific, pro-inflammatory response in HLA-DR4 transgenic mice in a dampening fashion.
van Lierop Mj et al., 2007 46	-MIA and HC gp-39 levels in SF and ST were determined by enzyme-linked immunosorbent assay and immunohistochemistry. -IL-2 production of T cell hybridomas generated by immunizing DR4-transgenic mice with HC gp-39 or MIA were used to study presentation of MIA and HC gp-39 by SF cells.	• HC gp-39 and MIA were found in the SF and ST of patients with arthritis, and they were mainly presented by HLA-DR myeloid dendritic cells and B cells.
Li et al., 2017 47	-In vivo study using recombinant human YKL-40.	• YKL-40 activates the FAK/PI3K/Akt pathway, thereby inducing the production of IL-18 in osteoblasts and the inhibition of miR-590-3p. • Stimulation of EPC angiogenesis, subsequently promoting inflammation, a process critical to the pathogenesis of RA.
Kazakova et al., 2017 48	-Analysis of serum and synovial YKL-40, TNF-α, IL-6, and IL-1β samples obtained from 39 RA patients using ELISA assay.	• IL-1β is the most important factor involved in the inflammatory process in RA • TNF-α might induce secretion of YKL-40 from chondrocytes. • Serum and synovial YKL-40, IL-1β, and TNF-α levels are strongly correlated, suggesting that these molecules together play an important role in RA pathogenesis and disease activity. • No correlation exists between YKL-40 and IL-6 levels.
Nielsen et al., 2011 49	-Analysis of serum and whole blood samples from 308 RA patients and 605 healthy blood donors.	• The g.-131 (C > G) promoter SNP in the *CHI3L1* gene is strongly associated with the serum concentration of YKL-40 in both RA patients and in healthy controls. • The g.-131 (C > G) promoter SNP does not appear to be a direct risk factor of RA itself.
Baeten D et al., 2004 50	-Synovial biopsy samples were gathered for diagnostic evaluation from 154 patients.	• HLA-DR shared epitope (HC gp-39(263-275) complexes) and crystal deposition had positive predictive values for diagnosis of >90% in patients for atypical RA patients.
Brahe et al., 2018 53	-Two investigator-initiated RCTs performed on treatment-naïve ERA patients.	• sIL-6, sYKL-40, and pVEGF levels were significantly correlated with DAS28 at baseline. • They did not appear to be predictive of either clinical remission or radiographic progression.
Väänänen et al., 2017 54	-RCT (NEO-RACo study) on 99 ERA patients undergoing DMARD therapy for 26 weeks.	• Baseline pYKL-40 levels in ERA patients showed a positive correlation with disease activity as well as with IL-6 and MMP-3 levels. • During DMARD treatment, YKL-40 levels decreased significantly, which appears to be directly related to the anti-rheumatic effect of DMARDs. • YKL-40 could potentially be used as a biomarker of disease activity in both ERA and RA patients undergoing active DMARD treatment.
Turkyilmaz et al., 2013 55	-Cross-sectional study of 42 ERA patients and 35 healthy subjects. -Evaluation of arterial stiffness by cf-PWV and IMT-C by carotid ultrasonography.	• The patients' sYKL-40 levels were strongly correlated with both cf-PWV and DAS28. • sYKL-40 levels could reflect early atherosclerosis, a major factor that contributes to mortality, as well as disease activity in ERA patients.
Kazakova et al., 2012 56	-Comparison of YKL-40 levels in serum and synovial fluid and ultrasonographic findings obtained from 25 RA patients and 40 healthy subjects.	• The Bulgarian RA patients were found to have significantly higher sYKL-40 levels than the healthy subjects. • There is a positive correlation between YKL-40 in serum and synovial fluid and sonographic parameters.
Knudsen et al., 2008 56	-Analysis of biochemical measurements, radiographs, and MRI images obtained from 51 ERA patients and 21 PA patients.	• pIL-6, sYKL-40, pVEGF, CRP, and ESR were elevated in RA patients but not in PA patients. • Only pIL-6 was related to treatment response and progressive erosive disease in ERA; sYKL-40 showed no association.
Bakker M et al., 2012 57	-Twelve biomarkers, including YKL-40, were used to calculate the MBDA score of RA patients.	• YKL-40 was one of the markers of the MBDA score designed to measure RA disease activity. • The MBDA score showed a significant correlation in measuring disease activity of RA.
Tanaka Y et al., 2014 51	-Analysis of HC gp-39 serum levels and mRNA expressions using ELISA and RT-PCR in GPI-induced arthritis.	• On the early phase of GPI-induced arthritis, HC gp-39 mainly showed in CD4+CD25+FoxP3+ Treg cells. • Addition of recombinant HC gp-39 blocked GPI-specific T cell proliferation and cytokine production, which means HC gp-39 in CD4+ T cells might play a regulatory role in early RA.
Knudsen et al., 2006 58	-1-year pilot study of sYKL-40 in 20 RA patients undergoing 52 weeks of infliximab and concomitant methotrexate therapy	• High baseline pIL-6 is significantly related to radiographic joint destruction progression. • It is unknown whether sYKL-40, independent of ESR, can provide information on joint destruction and disease activity in RA patients being treated with infliximab.
Syversen et al., 2009 59	-Cohort study of 238 RA patients followed for 10 years. -Analysis of serum samples and radiograph data.	• In contrast to sCTX-1 levels, sYKL-40 levels are weakly associated with radiographic progression and inflammation in RA. • sYKL-40 will likely be much less useful as a prognostic marker of RA.
Landewé RB et al., 2010 60	-A phase II, 13-week multicenter, double-blinded RCT on patients with RA to receive either intranasal applications of placebo. or fully human, recombinant HC gp-39 (Org39141) in differential doses once a week.	• Intranasal administration of Org39141 was proven to be safe but showed no efficacy over placebo.
Wolvers DA et al., 1999 61	-Mouse model was intra-nasally administered with OVA and HC gp-39 for tolerance induction. -Lymph nodes were surgically removed and re-transplanted to see importance of lymph nodes in tolerance induction.	• Certain lymph nodes that drain the nasal mucosa are essential in intranasal tolerance induction of model Ag (OVA) and HC gp-39. • Therapeutic potential of intranasal administration of HC gp-39 in RA.

Abbreviations: RA: rheumatoid arthritis; PBMC: peripheral blood mononuclear cell; HC gp-39: human cartilage glycoprotein-39; PCR: polymerase chain reaction; RT-PCR: reverse transcription polymerase chain reaction; ELISA: enzyme-linked immunosorbent assay; ST: synovial tissue; DMARD: disease-modifying anti-rheumatic drugs; HLA: human leukocyte antigen; OA: osteoarthritis; AVP: arginine vasopressin; PTHrP: parathyroid hormone-related peptide; SLE: systemic lupus erythematosus; IBD: inflammatory bowel disease; ANOVA: analysis of variance; sYKL-40: serum YKL-40; ERA: early rheumatoid arthritis; MHC: major histocompatibility complex; Ag: antigen; mAb: monoclonal antibody; TCR: T-cell receptor; sIGF-I: serum insulin-like growth factor I; sIL-: serum interleukin; CRP: C-reactive protein; RIA: radioimmunoassay; TNF: tumor necrosis factor; pIL-: plasma interleukin; pVEGF: plasma vascular endothelial growth factor; CII: type II collagen; MIA: melanoma inhibitory activity; SF: synovial fluid; IL-: interleukin; FAK: focal adhesion kinase; PI3K: phosphoinositide 3-kinase; Akt: protein kinase B; miR: microRNA; EPC: endothelial progenitor cell; SNP: single nucleotide polymorphism; RCT: randomized controlled trial; DAS28: disease activity score (28 joints); pYKL-40: plasma YKL-40; MMP-3: matrix metalloproteinase-3; cf-PWV: carotid to femoral pulse wave velocity; IMT-C: carotid intima media thickness; MRI: magnetic resonance imaging; PA: polyarthritis; ESR: erythrocyte sedimentation rate; MBDA: multi-biomarker disease activity; GPI: glucose-6-phosphate isomerase; FoxP3: forkhead box protein 3; Treg cell: regulatory T cell; sCTX-1: serum collagen cross-linked C-telopeptide; OVA: ovalbumin.

**Table 2:** List of studies on the relationship between psoriasis and YKL-40 (chitinase-3-like protein-1, human cartilage glycoprotein-39).

Authors, year	Study design	Main study findings
***Positive correlation with YKL-40***		
Imai Y et al., 2011. 64	-62 psoriasis patients and 21 controls were studied. -Serum YKL measured using ELISA.	• PV patients have 3-times higher YKL-40 than controls while GPP patients have higher YKL-40 levels than PV patients. • Involvement in joint inflammation or more severe inflammatory psoriasis patients have higher YKL-40.
Imai Y et al., 2013. 65	-Diagnosis YKL-40 in 18 PsA patients and 29 controls based on CASPAR criteria using enzyme-linked immunoassay kit.	• In PsA patients, serum YKL-40 levels may be a useful biomarker reflecting severity of skin lesions.
Ahmed S et al., 2015. 66	-48 psoriasis patients and 30 controls were evaluated using high-resolution PDUS. -sYKL-40 levels were measured using ELISA.	• Compared to controls, an obvious elevation was detected in serum YKL-40 levels in psoriasis patients. (p<0.001). • YKL-40 could be applied for assessing angiogenesis and disease activity in psoriasis patients. • PDUS is a good non-invasive tool for detecting early synovial changes in psoriasis patients and evaluating angiogenesis in PsA patients.
Salomon J et al., 2017. 67	-Blood of 55 psoriatic patients were taken to check the serum of YKL-40, CRP, ESR, etc.	• YKL-40 could be a useful marker of inflammation in psoriasis and might indicate psoriatic patients with systemic inflammation.
Salomon J et al., 2018. 68	-Blood of 42 psoriatic arthritis patients were taken to check the serum of YKL-40, CRP, ESR, etc.	• YKL-40 might be a potential marker for diagnosis and monitoring in psoriatic arthritis patients.
Baran A et al., 2018. 69	-Comparison the blood of 37 psoriasis patients,15 healthy controls before and after therapy.	• YKL-40 might be a marker of psoriasis but it is not useful to assess the metabolic conditions, severity and efficacy of treatment.
Abu El-Hamd M et al., 2018. 70	-A cross-sectional case-control included 30 psoriasis vulgaris patients and 20 healthy individuals. -After NB-UVB phototherapy, assessing the serum levels of YKL-40 of them.	• NB-UVB phototherapy, an essential method for psoriasis vulgaris patients, resulted in decreasing the serum levels of YKL-40.
Alpsoy S et al., 2014. 71	-Measured 60 psoriasis patients and 50 controls' demographic and biochemical parameters, C3, C4, D-dimer, CRP, fibrinogen and YKL-40.	• In psoriasis patients, CRP, YKL-40 and PASI score showed positive correlation in increase cIMT and impaired aortic elasticity. • Levels of increased C3 and fibrinogen showed negative correlation with aortic strain and compliance.
Erfan G et al., 2015. 72	-60 psoriasis patients and 30 healthy controls were evaluated for endothelial function and serum YKL-40.	• The increased level of YKL-40 in psoriasis might be related to ED. • YKL-40 also can be valuable for managing cardiovascular diseases in RP psoriasis patients above 40 ages.
***Negative or none correlation with YKL-40***		
Ataseven A et al., 2016. 73	-56 psoriatic patients were included in the study. -Vaspin, VAP-1, YKL-40 and hs-CRP levels were evaluated.	• Vaspin and VAP-1 can be used as markers of psoriasis. • Difference of sYKL-40 between psoriatic patients and the control group was no significant.
Jensen P et al., 2013. 74	-Measured YKL-40, hs-CRP and PASI in 48 psoriasis patients and repeated measurements after 4-6 weeks of MTX treatment in a subgroup of 14 patients. -Measured YKL-40, hs-CRP in 42 PsA patients at initiation of study and during 48 weeks of adalimumab treatment	• Elevation of YKL-40 has been well-defined in those of patients with PsA, not psoriasis. • Decrease of YKL-40 in PsA patients who responded to treatment. As a result, YKL-40 could be a useful biomarker to monitor PsA patients to check the effect of treatment with TNF- α inhibitors.

Abbreviations: ELISA: enzyme linked immunosorbent assay; PV: psoriasis vulgaris; GPP: generalized pustular psoriasis; PsA; psoriatic arthritis; CASPAR: classification for psoriatic arthritis; PDUS: power doppler ultrasound; CRP: C-reactive protein; ESR: Erythrocyte sedimentation rate; NB-UVB: Narrow-band Ultraviolet B; PASI: psoriasis area and severity index; cIMT: carotid intima-media thickness; ED: Endothelial dysfunction; RP: Risk-positive; VAP: Vascular adhesion protein; hs-CRP: high sensitivity C-reactive protein; MTX: methotrexate; TNF- α: tumor necrosis factor-α.

**Table 3:** List of studies on the relationship between systemic lupus erythematosus and YKL-40 (chitinase-3-like protein-1, human cartilage glycoprotein-39).

Authors, year	Study design	Main study findings
Vos K et al., 2000 36	-Comparison of plasma HC gp-39 levels in 50 RA, 51 OA, 24 SLE, 26 IBD patients and 49 healthy controls by one-way ANOVA.	• SLE patients have higher serum levels of HC gp-39 than healthy controls but lower than RA patients.
Dominika Wcisło Dziadecka et al., 2009 79	-Comparison of serum HC gp-39 levels in 25 SLE patients and 22 healthy controls by immunosorbent assay (METRA YKL-40 kit).	• Mean serum levels of HC gp-39 were almost half in controls than in lupus patients. • Only HC gp-39 levels and γ-globulin showed positive correlation. (r=0.40, p<0.05). • No correlation between HC gp-39 and age, BMI, duration of symptoms, serum CRP, ANA titer, ESR, disease activity measured with the SLEDAI. • No difference in serum HC gp-39 between the subgroups of patients with the SLEDAI<30 and those with the SLEDAI>30. • Although HC gp-39 thought to be an index of cartilage damage initially, it should be considered as an index of chondrocyte activation due to subsequent studies.
Vos K et al., 2000 80	-Growth with 5 different HC gp-39 derived peptides followed by measuring multiplication of PBMC as well as recording disease activity score in RA, SLE, IBD, OA and healthy controls.	• In autoimmune diseases including SLE, HC gp-39 derived peptides are the objects of T cell immunity. • The T-cell response to the various HC gp-39 derived peptide was low in the SLE patients.

Abbreviations: HC gp-39: human cartilage glycoprotein-39; RA: rheumatoid arthritis; OA: osteoarthritis; SLE: systemic lupus erythematosus; IBD: inflammatory bowel disease; ANOVA: analysis of variance; BMI: body mass index; CRP: C-reactive protein; ANA: anti-nuclear antibody; ESR: erythrocyte sedimentation rate; SLEDAI: systemic lupus erythematosus disease activity index; PBMC: Peripheral blood mononuclear cell.

**Table 4:** List of studies on the relationship between Behçet disease and YKL-40 (chitinase-3-like protein-1, human cartilage glycoprotein-39).

Authors, year	Study design	Main study findings
Seo J et al., 2016 84	-Comparison of plasma levels of YKL-40 in 112 Behcet's disease patients and 45 healthy volunteers.	• Serum YKL-40 might be a provider of the pathophysiology of Behcet's disease and useful marker for monitoring Behcet's disease patients.
Bilen H et al, 2016 85	-Comparison of plasma levels of chitinase-3-like 1 protein and its association with malondialdehyde in 51 Behcet's disease patients and 28 healthy controls by SPSS 20.	• Chitinase-3-like 1 protein might be associated with Behcet's disease. • The levels of malondialdehyde had no significant correlation with Chitinase-3-like 1 protein.

Abbreviations: SPSS: statistical package for social science.

**Table 5:** List of studies on the relationship between Inflammatory bowel disease and YKL-40 (chinitinase-3-like protein-1, human cartilage glycoprotein-39).

Authors, year	Study design	Main study findings
***Strong Correlation between YKL-40 and disease activity***		
Koutroubakis I. E et al., 2003 86	-Detection of sYKL-40 values in 94 UC, 85 CD, 23 non-IBD intestinal inflammation patients and 70 healthy controls by using ELISA.	• Mean sYKL-40 concentrations increased in UC and CD patients than in healthy controls (*P*<0.0001, respectively), but not significantly higher than in non-IBD intestinal inflammation patients. • YKL-40 levels increased as disease activity (*r*=0.29, *P*=0.005) and CRP (*r*=0.27, *P*=0.01) increased in both UC and CD patients. • Localization also attributed to higher YKL-40 levels as CD in ileum showed higher levels of YKL-40 than in ileocolon or colon. • Stenosis didn't make differences in YKL-40 levels.
Erzin Y et al., 2018. 87	-Serum YKL‐40 levels in 41 CD patients (including 12 patients with strictures) and 46 healthy controls were studied using multivariate regression analysis.	• YKL‐40 values in CD were highly detected in the healthy controls (*P*=0.000) and increased as disease activity increased (r=0.681, p=0.000). • Strictures in CD also showed contributions to higher serum YKL‐40 values (*r*=0.457, *P*=0.003). • YKL‐40 values could be a biomarker of clinical activity and formation of strictures in CD patients.
Vind I et al., 2003. 88	-Serum YKL-40 concentrations, albumin, CRP and leucocytes in 164 UC, 173 CD patients and 245 healthy controls were analyzed. -Disease activities in UC and CD were assessed by SCCAI and H-B scores.	• YKL-40 concentrations in severe active UC increased than in inactive UC and healthy controls(p<0.001). • Median YKL-40 concentrations in UC patients and clinical activity were correlated. • YKL-40 concentrations in severe active CD were also highly detected than in healthy controls(p<0.001), but not significantly higher than in inactive CD patients. • Median YKL-40 levels and H-B index in CD patients were not correlated. • Correlations between YKL-40 levels, albumin, CRP and leukocytes were very low.
Ytting H et al., 2005. 89	-YKL-40 levels, blood cell count, albumin and SSCAI was assessed in a SS patient.	• Serum YKL-40 concentrations increased as SCCAI and CRP levels increased during disease course. • YKL-40 was the highest when the SCCAI score was highest.
Bernardi D et al., 2003. 90	-YKL-40, CRP, SAA levels are assessed in 29 PsA, 66 IBD (36 CD and 30 UC), and 76 JIBD (44 CD and 32 UC).	• YKL-40 levels were highly detected in JIBD patients (*P* =0.000003) than in IBD patients, but there were no differences for CRP and SAA values. • No differences between JIBD and PsA patients for YKL-40, CRP, or SAA levels.
Vos K et al., 2000 91	-Effect of HC gp-39 derived peptide on PBMC proliferation was measured in RA, SLE, IBD, OA patients and healthy controls.	• HC gp-39-derived peptide makes PBMC proliferate more in inflammatory conditions like RA, IBD, OA than in healthy controls. • Level of peptide correlated with RA disease activity while it doesn't in IBD or SLE.
Buisson A et al., 2016. 92	-Fecal calprotectin and CHI3L1 was measured in 86 adult IBD patients and compared to CDEIS score in CD patients or MES in UC patients, evaluating both level as biomarker of activity in IBD.	• Both fecal CHI3L1 and calprotectin levels correlated with endoscopic activity score in CD and UC. • Fecal CHI3L1 seem to be reliable biomarker in assessing endoscopic activity of IBD.
Aomatsu T et al., 2011 93	-Analyzed the level of CHI3L1 and calprotectin in fecal samples from pediatric UC, CD patients and healthy control by ELISA.	• Active IBD patients' fecal CHI3L1 levels were higher compared with healthy control, and were correlated with disease activity.
Punzi L et al., 2003. 94	-Comparison of serum HC gp39 levels and CRP in 58 IBD-nonA, 63 IBD-A, and in 20 healthy controls. IBD patients were also divided into aIBD and naIBD.	• The serum HC gp39 values in IBD-A patients were highly detected than in controls (p<0.01) and IBD-nonA (p<0.001) patients. • Otherwise, the values between controls and IBD-nonA were not different. • Correlation between the serum HC gp-39 levels and NAJ (r=0.6, p<0.001). • HC gp39 could be a biomarker of arthropathy in IBD. • The serum CRP levels were high in IBD-A(p<0.01) and IBD-nonA(p<0.05) than in the controls. • CRP levels were highly detected in aIBD-nonA than in naIBD-nonA (p<0.05), the levels between aIBD-A and naIBD-A were not different.
Deutschmann C et al., 2019. 95	-Analyzed IgG, IgA, and sIgA to CHI3L1 by ELISAs in 331 IBD patients (110 CD, 95 UC, 126 CeD) and 86 healthy controls.	• Higher level of IgG, IgA, sIgA to CHI3L1 was detected in CD patients compared with UC, CeD and healthy controls. • Glycosylhydrolase family member CHI3L1 is novel neutrophil autoantigen target.
***Relationship between YKL-40 and development of cancer in IBD.***		
Chen CC et al., 2011. 96	-Compared CHI3L1expression of colonic samples from UC patients and healthy control. -Analyzed the effect of CHI3L1 on CECs to malignant change in inflammatory condition.	• High expression of CHI3L1 was shown in IBD patients with neoplasia compared with that in healthy control or IBD patients without neoplasia. • CHI3L1 seems to promote tumor progression in inflammatory conditions by inducing growth, proliferation, migration of CECs.
Low D et al., 2015. 97	-Compared incidence of CAC in CHI3L1 knockout mice and wild type mice both treated with AOM/DSS.	• In chronic intestinal inflammatory condition, highest CHI3L1 expression was found and is critical for IEC survival and proliferation, contributing tumor formation in colitis. • Fecal CHI3L1 level can be used as marker of tumor progression in IBD patients. • CHI3L1 competitively inhibit S100A9, and balance of these two molecules determine IECs to proliferate or to do apoptosis.
Low D et al., 2015. 98	-After inducting tumor by using azoxymethane in MOLF/EiJ mouse that overexpress colonic epithelial CHI3L1, immunohistochemical, microscopic, statistical analysis were conducted.	• High CHI3L1 level is related to spontaneous development of polypoid nodule and colonic immune cell infiltration and makes azoxymethane induce colorectal cancer more easily.
Ma JY et al., 2014. 99	-Macroscopic, histologic, molecular biologic analysis was conducted in 5 groups of Balb/c mice (Control/ CAC/ CAC + caffeine/ Colitis/ Colitis +caffeine).	• In colon carcinogenesis, CHI3L1 increase risk of tumor by production of reactive oxygen species. • Caffeine decreases the risk of tumor by reducing oxidative DNA damage.

* YKL-40 is positively correlated in all related IBD papers. Abbreviations: UC: Ulcerative colitis; CD: Crohn's disease; IBD: Inflammatory bowel disease; ELISA: Enzyme-linked immunosorbent assay; CRP: C reactive protein; SCCAI: Simple Clinical Colitis Activity Index; H-B: Harvey-Bradshaw; SS: Sweet's syndrome (Extraintestinal manifestation of inflammatory bowel disease); SAA: Serum amyloid A; PsA: psoriatic arthritis; JIBD: Joint involvement in inflammatory bowel disease; HC gp-39: Human cartilage glycoprotein 39; PBMC: peripheral blood mononuclear cell; RA: Rheumatoid arthritis; SLE: Systemic lupus erythematosus; OA: osteoarthritis; CHI3L1: Chitinase-3-like protein 1; CDEIS: Crohn's disease Endoscopic Index of Severity; MES: Mayo endoscopic subscore; IBD-nonA: Inflammatory bowel disease without arthritis; IBD-A: Inflammatory bowel disease with arthritis; aIBD: active inflammatory bowel disease; naIBD; non active inflammatory bowel disease; NAJ: Number of affected joints; Ig: immunoglobulin; sIgA: secretory immunoglobulin A; CeD: celiac disease; CECs: colonic epithelial cells; CAC: colitis associated cancer; AOM/DSS: Azoxymethane/dextran sulphate sodium; IEC: intestinal epithelial cell; S100A9: S100 calcium-binding protein 9.

**Table 6:** List of other studies on YKL-40 (chitinase-3-like protein-1, human cartilage glycoprotein-39) as biomarker of human disease with autoimmune mechanism.

Authors, year	Study design	Main study findings
Dönder et al., 2021^100^	Serum levels of YKL-40 in three groups: 1) patients with CIS (n = 20); 2) patients with relapsing-remitting MS (RRMS; n = 39); and 3) healthy individuals (n = 35).	• Median serum YKL-40 level was 20.2 ng/mL in the patients with CIS, 22.7 ng/mL in the patients with RRMS and 11.0 ng/mL in the control group (p < 0.001) • Serum YKL-40 levels in the patients with RRMS were correlated with the patients' expanded disability status scale scores and ages (p < 0.05)
Valentin et al. 2021 101	Serum levels of YKL-40 were examined of forty female patients with SS (26 with diffuse cutaneous (dcSSc) and 14 with limited cutaneous SSc (lcSSc)) and 14 healthy female controls were enrolled in this cross-sectional study.	• YKL-40 serum levels were significantly higher in patients compared to controls (p=0.0042). In contrary, miR-214 expression in plasma of SSc patients was significantly downregulated compared to controls (p=0.0058).
Krzysztof et al., 2015 102	The study group comprised 167 patients, including 116 women and 51 men aged 18-88 years with chronic asthma to investigate the role of YKL-40 as a possible marker of asthma.	• Significantly higher YKL-40 in subgroup with poor control of symptoms and exacerbations (91.8 ± 57.1 ng/ml) compared to stable asthmatics (59.6 ± 50.8 ng/ml; p=0.001) as well as in atopic compared to non-atopic asthmatics (77.2 ± 53.9 vs. 61.1 ± 57.8 ng/ml; p=0.001).
Llorens et al., 2017 103	CSF YKL-40 levels were measured in a cohort of 288 individuals, including (NC) and patients diagnosed with different types of dementia	• Compared to neurological controls, increased YKL-40 levels were detected in sCJD (p < 0.001, AUC = 0.92) and AD (p < 0.001, AUC = 0.77) but not in vascular dementia (VaD) (p > 0.05, AUC = 0.71) or in DLB/Parkinson's disease dementia (PDD) (p > 0.05, AUC = 0.70).

Abbreviations: chronically isolated syndrome; CIS, multiple sclerosis; MS, systemic sclerosis; SS, polymyositis/dermatomyositis; PM/DM, Neuromyelitis optica spectrum disorders; NMOSD, Alzheimer's disease; AD, modified Rankin Scale; mRS. Cerebrospinal fluid; CSF, Creutzfeldt-Jakob disease; sCJD, dementia with Lewy bodies; DLB.

## References

[1] Rehli M, Krause SW, Andreesen R (1997). Molecular characterization of the gene for human cartilage gp-39(CHI3L1), a member of the chitinase protein family and marker for late stages of macrophage differentiation. Genomics.

[2] Shackelton LM, Mann DM, Millis AJ (1995). Identification of a 38-kDa heparin-binding glycoprotein (gp38k) in differentiating vascular smooth muscle cells as a member of a group of proteins associated with tissue remodeling. J Biol Chem.

[3] Hakala BE, White C, Recklies AD (1993). Human cartilage gp-39, a major secretory product of articular chondrocytes and synovial cells, is a mammalian member of a chitinase protein family. J Biol Chem.

[4] Johansen JS, Jensen BV, Roslind A, Nielsen D, Price PA (2006). Serum YKL-40, a new prognostic biomarker in cancer patients?. Cancer Epidemiol Biomarkers Prev.

[5] Rathcke Camilla N, Henrik Vestergaard YKL-40-an emerging biomarker in cardiovascular disease and diabetes. Cardiovascular diabetology 8.1 (2009): 61.

[6] Hu B, Trinh K, Figueira WF, Price PA (1996). Isolation and sequence of a novel human chondrocyte protein related to mammalian members of the chitinase protein family. J Biol Chem.

[7] Hauschka PV, Mann KG, Price P, Termine JD (1986). Report of the Ad Hoc Committee on Nomenclature and Standards for Bone Proteins and Growth Factors. J Bone Miner Res.

[8] Cintin C, Johansen JS, Christensen IJ, Price PA, Sørensen S, Nielsen HJ (1999). Serum YKL-40 and colorectal cancer. Br J Cancer.

[9] Muszyński P, Groblewska M, Kulczyńska-Przybik A, Kułakowska A, Mroczko B (2017). YKL-40 as a Potential Biomarker and a Possible Target in Therapeutic Strategies of Alzheimer's Disease. Current Neuropharmacology.

[10] Johansen JS, Stoltenberg M, Hansen M, Florescu A, Hørslev-Petersen K, Lorenzen I, Price PA (1999). Serum YKL-40 concentrations in patients with rheumatoid arthritis: relation to disease activity. Rheumatology (Oxford).

[11] Faibish M, Francescone R, Bentley B, Yan W, Shao R (2011). A YKL-40-neutralizing antibody blocks tumor angiogenesis and progression: a potential therapeutic agent in cancers. Molecular Cancer Therapeutics.

[12] Morgante M, Di Munno O, Morgante D (1999). YKL 40: marker of disease activity in rheumatoid arthritis?. Minerva Med.

[13] Kzhyshkowska J, Gudima A, Moganti K, Gratchev A, Orekhov A (2016). Perspectives for Monocyte/Macrophage-Based Diagnostics of Chronic Inflammation. Transfus Med Hemother.

[14] Baeten D, Boots AM, Steenbakkers PG, Elewaut D, Bos E, Verheijden GF (2000). Human cartilage gp-39+,CD16+ monocytes in peripheral blood and synovium: correlation with joint destruction in rheumatoid arthritis. Arthritis Rheum.

[15] Kirkpatrick RB, Emery JG, Connor JR, Dodds R, Lysko PG, Rosenberg M (1997). Induction and expression of human cartilage glycoprotein 39 in rheumatoid inflammatory and peripheral blood monocyte-derived macrophages. Exp Cell Res.

[16] Harvey S, Weisman M, O'Dell J, Scott T, Visor MK, Swindlehurst C (1998). Chondrex: new marker of joint disease. Clinical chemistry.

[17] Lorand V, Balint Z, Komjati D, Ne meth B, Minier T, Kumanovics G (2016). Validation of disease activity indices using the 28 joint counts in systemic sclerosis. Rheumatology.

[18] Johansen J (2006). Studies on serum YKL-40 as a biomarker in diseases with inflammation, tissue remodelling, fibroses and cancer. Danish Medical Bulletin.

[19] Kzhyshkowska J, Gratchev A, Goerdt S (2007). Human Chitinases and Chitinase-Like Proteins as Indicators for Inflammation and Cancer. Biomarker Insights.

[20] van Bilsen JH, van Dongen H, Lard LR, van der Voort EI, Elferink DG, Bakker AM (2004). Functional regulatory immune responses against human cartilage glycoprotein-39 in health vs. proinflammatory responses in rheumatoid arthritis. Proc Natl Acad Sci USA.

[21] Hakala BE, White C, Recklies AD (1993). Human cartilage gp-39, a major secretory product of articular chondrocytes and synovial cells, is a mammalian member of a chitinase protein family. J Biol Chem.

[22] Verheijden GF, Rijnders AW, Bos E, Coenen-de Roo CJ, van Staveren CJ, Miltenburg AM (1997). Human cartilage glycoprotein-39 as a candidate autoantigen in rheumatoid arthritis. Arthritis Rheum.

[23] Patil NS, Hall FC, Drover S, Spurrell DR, Bos E, Cope AP (2001). Autoantigenic HCgp39 epitopes are presented by the HLA-DM-dependent presentation pathway in human B cells. The Journal of Immunology.

[24] Baeten D, Steenbakkers PG, Rijnders AM, Boots AM, Veys EM, De Keyser F (2004). Detection of major histocompatibility complex/human cartilage gp-39 complexes in rheumatoid arthritis synovitis as a specific and independent histologic marker. Arthritis & Rheumatism.

[25] Kavanaugh A, Genovese M (2003). Allele and antigen-specific treatment of rheumatoid arthritis: a double blind, placebo controlled phase 1 trial. The Journal of rheumatology.

[26] Petersson M, Bucht E, Granberg B, Stark A (2006). Effects of arginine-vasopressin and parathyroid hormone-related protein (1-34) on cell proliferation and production of YKL-40 in cultured chondrocytes from patients with rheumatoid arthritis and osteoarthritis. Osteoarthritis and Cartilage.

[27] Volck B, Price P, Johansen J, Sørensen O, Benfield T, Nielsen H (1998). YKL-40, a mammalian member of the chitinase family, is a matrix protein of specific granules in human neutrophils. Proceedings of the Association of American Physicians.

[28] Vos K, Steenbakkers P, Miltenburg A, Bos E, van den Heuvel M, van Hogezand R (2000). Raised human cartilage glycoprotein-39 plasma levels in patients with rheumatoid arthritis and other inflammatory conditions. Annals of the Rheumatic Diseases.

[29] Harvey S, Whaley J, Eberhardt K (2000). The relationship between serum levels of YKL-40 and disease progression in patients with early rheumatoid arthritis. Scandinavian Journal of Rheumatology.

[30] Sekine T, Masuko-Hongo K, Matsui T, Asahara H, Nishioka K, Kato T (2001). Recognition of YKL-39, a human cartilage related protein, as a target antigen in patients with rheumatoid arthritis. Annals of the Rheumatic Diseases.

[31] Steenbakkers PG, Baeten D, Rovers E, Veys EM, Rijnders AW, Meijerink J (2003). Localization of MHC class II/human cartilage glycoprotein-39 complexes in synovia of rheumatoid arthritis patients using complex-specific monoclonal antibodies. J Immunol.

[32] Matsumoto T, Tsurumoto T (2001). Serum YKL-40 levels in rheumatoid arthritis: correlations between clinical and laboratory parameters. Clinical and Experimental Rheumatology.

[33] Johansen J, Kirwan J, Price P, Sharif M (2001). Serum YKL-40 concentrations in patients with early rheumatoid arthritis: relation to joint destruction. Scandinavian Journal of Rheumatology.

[34] Johansen J, Stoltenberg M, Hansen M, Florescu A, Hørslev-Petersen K, Lorenzen I (1999). Serum YKL-40 concentrations in patients with rheumatoid arthritis: relation to disease activity. Rheumatology.

[35] Volck B, Johansen J, Stoltenberg M, Garbarsch C, Price P, Østergaard M (2001). Studies on YKL-40 in knee joints of patients with rheumatoid arthritis and osteoarthritis. Involvement of YKL-40 in the joint pathology. Osteoarthritis and Cartilage.

[36] Peltomaa R, Paimela L, Harvey S, Helve T, Leirisalo-Repo M (2001). Increased level of YKL-40 in sera from patients with early rheumatoid arthritis: a new marker for disease activity. Rheumatology International.

[37] Vos K, Miltenburg AM, Van Meijgaarden KE, van den Heuvel M, Elferink DG, van Galen PJ (2000). Cellular immune response to human cartilage glycoprotein-39 (HC gp-39)-derived peptides in rheumatoid arthritis and other inflammatory conditions. Rheumatology.

[38] den Broeder A (2002). Long term anti-tumour necrosis factor alpha monotherapy in rheumatoid arthritis: effect on radiological course and prognostic value of markers of cartilage turnover and endothelial activation. Annals of the Rheumatic Diseases.

[39] Combe B, Dougados M, Goupille P, Cantagrel A, Eliaou J, Sibilia J (2001). Prognostic factors for radiographic damage in early rheumatoid arthritis: A multiparameter prospective study. Arthritis & Rheumatism.

[40] Fusetti F, Pijning T, Kalk KH, Bos E, Dijkstra BW (2003). Crystal structure and carbohydrate-binding properties of the human cartilage glycoprotein-39. Journal of Biological Chemistry.

[41] Fikry EM, Gad AM, Eid AH, Arab HH (2019). Caffeic acid and ellagic acid ameliorate adjuvant-induced arthritis in rats via targeting inflammatory signals, chitinase-3-like protein-1 and angiogenesis. Biomedicine & Pharmacotherapy.

[42] Zandbelt MM, Houbiers JG, van den Hoogen FH, Meijerink J, van Riel PL, in't Hout J (2006). Intranasal administration of recombinant human cartilage glycoprotein-39. A phase I escalating cohort study in patients with rheumatoid arthritis. J Rheumatol.

[43] Knudsen L, Hetland M, Johansen J, Skjødt H, Peters N, Colic A (2009). Changes in Plasma IL-6, Plasma VEGF and Serum YKL-40 During Treatment with Etanercept and Methotrexate or Etanercept alone in Patients with Active Rheumatoid Arthritis despite Methotrexate Therapy. Biomarker Insights.

[44] Tsark EC, Wang W, Teng YC, Arkfeld D, Dodge GR, Kovats S (2002). Differential MHC class II-mediated presentation of rheumatoid arthritis autoantigens by human dendritic cells and macrophages. J Immunol.

[45] Boots AM, Hubers H, Kouwijzer M, den Hoed-van Zandbrink L, Westrek-Esselink BM, van Doorn C (2007). Identification of an altered peptide ligand based on the endogenously presented, rheumatoid arthritis-associated, human cartilage glycoprotein-39(263-275) epitope: an MHC anchor variant peptide for immune modulation. Arthritis Res Ther.

[46] van Lierop MJ, den Hoed L, Houbiers J, Vencovsky J, Ruzickova S, Krystufkova O (2007). Endogenous HLA-DR-restricted presentation of the cartilage antigens human cartilage gp-39 and melanoma inhibitory activity in the inflamed rheumatoid joint. Arthritis Rheum.

[47] Li T, Liu S, Huang Y, Huang C, Hsu C, Tsai C (2017). YKL-40-Induced Inhibition of miR-590-3p Promotes Interleukin-18 Expression and Angiogenesis of Endothelial Progenitor Cells. International Journal of Molecular Sciences.

[48] Kazakova M, Batalov A, Mateva N, Kolarov Z, Sarafian V (2017). YKL-40 and cytokines - a New Diagnostic Constellation in Rheumatoid Arthritis?. Folia Medica.

[49] Nielsen K, Steffensen R, Boegsted M, Baech J, Lundbye-Christensen S, Hetland M (2011). Promoter polymorphisms in the chitinase 3-like 1 gene influence the serum concentration of YKL-40 in Danish patients with rheumatoid arthritis and in healthy subjects. Arthritis Research & Therapy.

[50] Baeten D, Kruithof E, De Rycke L, Vandooren B, Wyns B, Boullart L (2004). Diagnostic classification of spondylarthropathy and rheumatoid arthritis by synovial histopathology: A prospective study in 154 consecutive patients. Arthritis & Rheumatism.

[51] Tanaka Y, Matsumoto I, Inoue A, Umeda N, Takai C, Sumida T (2014). Antigen-specific over-expression of human cartilage glycoprotein 39 on CD4+ CD 25+ forkhead box protein 3+ regulatory T cells in the generation of glucose-6-phosphate isomerase-induced arthritis. Clinical & Experimental Immunology.

[52] Brahe C, Dehlendorff C, Østergaard M, Johansen J, Ørnbjerg L, Hørslev-Petersen K (2018). Circulating serum interleukin-6, serum chitinase-3-like protein-1, and plasma vascular endothelial growth factor are not predictive for remission and radiographic progression in patients with early rheumatoid arthritis: post-hoc explorative and validation studies based on the CIMESTRA and OPERA trials. Scandinavian Journal of Rheumatology.

[53] Väänänen T, Vuolteenaho K, Kautiainen H, Nieminen R, Möttönen T, Hannonen P (2017). Glycoprotein YKL-40: A potential biomarker of disease activity in rheumatoid arthritis during intensive treatment with csDMARDs and infliximab. Evidence from the randomised controlled NEO-RACo trial. PLOS ONE.

[54] Turkyilmaz A, Devrimsel G, Kirbas A, Cicek Y, Karkucak M, Capkin E (2013). Relationship between pulse wave velocity and serum YKL-40 level in patients with early rheumatoid arthritis. Rheumatology International.

[55] Kazakova M, Batalov A, Deneva T, Mateva N, Kolarov Z, Sarafian V (2012). Relationship between sonographic parameters and YKL-40 levels in rheumatoid arthritis. Rheumatology International.

[56] Knudsen L, Klarlund M, Skjødt H, Jensen T, Ostergaard M, Jensen K (2008). Biomarkers of inflammation in patients with unclassified polyarthritis and early rheumatoid arthritis. Relationship to disease activity and radiographic outcome. The Journal of Rheumatology.

[57] Bakker M, Cavet G, Jacobs J, Bijlsma J, Haney D, Shen Y (2012). Performance of a multi-biomarker score measuring rheumatoid arthritis disease activity in the CAMERA tight control study. Annals of the Rheumatic Diseases.

[58] Knudsen L, Østergaard M, Baslund B, Narvestad E, Petersen J, Nielsen H (2006). Plasma IL-6, plasma VEGF, and serum YKL-40: relationship with disease activity and radiographic progression in rheumatoid arthritis patients treated with infliximab and methotrexate. Scandinavian Journal of Rheumatology.

[59] Syversen S, Goll G, van der Heijde D, Landewé R, Gaarder P, Ødegård S (2009). Cartilage and Bone Biomarkers in Rheumatoid Arthritis: Prediction of 10-year Radiographic Progression. The Journal of Rheumatology.

[60] Landewé RB, Houbiers JG, Van den Bosch F, in't Hout J, Verschueren PC, Meijerink JH (2010). Intranasal administration of recombinant human cartilage glycoprotein-39 as a treatment for rheumatoid arthritis: a phase II, multicentre, double-blind, randomised, placebo-controlled, parallel-group, dose-finding trial. Ann Rheum Dis.

[61] Wolvers DA, Coenen-de Roo CJ, Mebius RE, van der Cammen MJ, Tirion F, Miltenburg AM (1999). Intranasally induced immunological tolerance is determined by characteristics of the draining lymph nodes: studies with OVA and human cartilage gp-39. J Immunol.

[62] Menter A, Gottlieb A, Feldman SR, Van Voorhees AS, Leonardi CL, Gordon KB (2008). Guidelines of care for the management of psoriasis and psoriatic arthritis: Section 1. Overview of psoriasis and guidelines of care for the treatment of psoriasis with biologics. J Am Acad Dermatol.

[63] Boehncke WH, Schön MP (2015). Psoriasis. Lancet.

[64] Imai Y, Tsuda T (2011). YKL-40 (chitinase 3-like-1) as a biomarker for psoriasis vulgaris and pustular psoriasis. Journal of dermatological science.

[65] Imai Y, Aochi S (2013). YKL-40 is a serum biomarker reflecting the severity of cutaneous lesions in psoriatic arthritis. The Journal of dermatology.

[66] Ahmed SF, Attia EAS (2015). Serum YKL-40 in psoriasis with and without arthritis; correlation with disease activity and high-resolution power Doppler ultrasonographic joint findings. Journal of the European academy of dermatology and venereology.

[67] Salomon J, Matusiak Ł (2017). Chitinase-3-like protein 1 (YKL-40) is a new biomarker of inflammation in psoriasis. Mediators of inflammation.

[68] Salomon J, Matusiak Ł (2018). Chitinase-3-like protein 1 (YKL-40) is a biomarker of severity of joint involvement in psoriatic arthritis. Advances in Dermatology and Allergology/Postȩpy Dermatologii i Alergologii.

[69] Baran A, Myśliwiec H (2018). Serum YKL-40 as a potential biomarker of inflammation in psoriasis. Journal of Dermatological Treatment.

[70] Abu El-Hamd M, Adam El Taieb M (2018). Serum YKL-40 in patients with psoriasis vulgaris treated by narrow-band UVB phototherapy. Journal of Dermatological Treatment.

[71] Alpsoy S, Akyuz A (2014). Atherosclerosis, some serum inflammatory markers in psoriasis. Giornale italiano di dermatologia e venereologia: organo ufficiale, Societa italiana di dermatologia e sifilografia.

[72] Erfan G, Guzel S (2015). Serum YKL-40: a potential biomarker for psoriasis or endothelial dysfunction in psoriasis?. Molecular and cellular biochemistry.

[73] Ataseven A, Kesli R (2016). Novel inflammatory markers in psoriasis vulgaris: vaspin, vascular adhesion protein-1 (VAP-1), and YKL-40. Giornale italiano di dermatologia e venereologia: organo ufficiale, Societa italiana di dermatologia e sifilografia.

[74] Jensen P, Wiell C (2013). Plasma YKL-40: a potential biomarker for psoriatic arthritis?. Journal of the European Academy of Dermatology and Venereology.

[75] Takahashi H, Tsuji H (2010). Serum cytokines and growth factor levels in Japanese patients with psoriasis. Clin Exp Dermatol.

[76] Kaul A, Gordon C, Crow MK, Touma Z, Urowitz MB, Vollenhoven RV (2016). Systemic lupus erythematosus. Nat Rev Dis Primers.

[77] Tsokos GC (2011). Systemic lupus erythematosus. N Engl J Med.

[78] Illei GG, Tackey E, Lapteva L, Lipsky PE (2004). Biomarkers in systemic lupus erythematosus. Arthr Rheum.

[79] Dominika Wcisło- Dziadecka, Anna Kotulska, Ligia Brzezińska- Wcisło, Eugeniusz J (2009). Kucharz, Anna Lis- Święty, Grażyna Kamińska- Wiciorek. Serum human cartilage glycoprotein- 39 levels in patients with systemic lupus erythematosus. Polskie Archiwum Medycyny Wewnetrznej.

[80] Vos K, Miltenburg AM, Van Meijgaarden KE, van den Heuvel M, Elferink DG, van Galen PJ (2000). Cellular immune response to human cartilage glycoprotein-39 (HC gp-39) derived peptides in rheumatoid arthritis and other inflammatory conditions. Rheumatology.

[81] Sakane T, Takeno M, Suzuki N, Inaba G (1999). Behçet's disease. N Engl J Med.

[82] Türsen U (2012). Pathophysiology of the Behçet's disease. Patholog Res Int.

[83] Kapsimali VD, Kanakis MA, Vaiopoulos GA, Kaklamanis PG (2010). Etiopathogenesis of Behçet's disease with emphasis on the role of immunological aberrations. Clin Rheumatol.

[84] Seo J, Ahn Y (2016). Clinical significance of serum YKL-40 in Behçet disease. British Journal of Dermatology.

[85] Bilen H, Altinkaynak K (2016). Serum YKL-40 and MDA levels in Behçet disease. J Pak Med Assoc.

[86] Koutroubakis IE, Petinaki E (2003). Increased serum levels of YKL-40 in patients with inflammatory bowel disease. International journal of colorectal disease.

[87] Erzin Y, Uzun H (2008). Serum YKL-40 as a marker of disease activity and stricture formation in patients with Crohn's disease. Journal of gastroenterology and hepatology.

[88] Vind I, Johansen JS (2003). Serum YKL-40, a potential new marker of disease activity in patients with inflammatory bowel disease. Scandinavian journal of gastroenterology.

[89] Ytting H, Vind I (2005). Sweet's syndrome-an extraintestinal manifestation in inflammatory bowel disease. Digestion.

[90] Bernardi D, Podswiadek M (2003). YKL-40 as a marker of joint involvement in inflammatory bowel disease. Clinical chemistry.

[91] Vos K, Miltenburg AMM (2000). Cellular immune response to human cartilage glycoprotein-39 (HC gp-39)-derived peptides in rheumatoid arthritis and other inflammatory conditions. Rheumatology.

[92] Buisson A, Vazeille E (2016). Faecal chitinase 3-like 1 is a reliable marker as accurate as faecal calprotectin in detecting endoscopic activity in adult patients with inflammatory bowel diseases. Alimentary pharmacology & therapeutics.

[93] Aomatsu T, Imaeda H (2011). Faecal chitinase 3-like-1: a novel biomarker of disease activity in paediatric inflammatory bowel disease. Alimentary pharmacology & therapeutics.

[94] Punzi L, Podswiadek M (2003). Serum human cartilage glycoprotein 39 as a marker of arthritis associated with inflammatory bowel disease. Annals of the rheumatic diseases.

[95] Deutschmann C, Sowa M (2018). Identification of Chitinase-3-like protein 1 as a novel neutrophil antigenic target in Crohn's disease. Journal of Crohn's and Colitis.

[96] Chen CC, Pekow J (2011). Chitinase 3-like-1 expression in colonic epithelial cells as a potentially novel marker for colitis-associated neoplasia. The American journal of pathology.

[97] Low D, Subramaniam R (2015). Chitinase 3-like 1 induces survival and proliferation of intestinal epithelial cells during chronic inflammation and colitis-associated cancer by regulating S100A9. Oncotarget.

[98] Low D, DeGruttola AK (2015). High Endogenous Expression of Chitinase 3-Like 1 and Excessive Epithelial Proliferation with Colonic Tumor Formation in MOLF/EiJ Mice. PloS one.

[99] Ma JY, Li RH (2014). Increased expression and possible role of chitinase 3-like-1 in a colitis-associated carcinoma model. World Journal of Gastroenterology: WJG.

[100] Dönder A, Özdemir HH (2021). Serum YKL-40 levels in patients with multiple sclerosis. Arq Neuropsiquiatr.

[101] Gao MZ, Wei YY, Xu QW, Ji R, Han ZJ, Jiang TW (2019). Elevated serum YKL-40 correlates with clinical characteristics in patients with polymyositis or dermatomyositis. Ann Clin Biochem.

[102] Specjalski K, Chełmińska M, Jassem E (2015). YKL-40 protein correlates with the phenotype of asthma. Lung.

[103] Llorens F, Thüne K, Tahir W, Kanata E, Diaz-Lucena D, Xanthopoulos K (2017). YKL-40 in the brain and cerebrospinal fluid of neurodegenerative dementias. Mol Neurodegener.

